# Structure of (*R*,*R*)-4-bromo-2-{4-[4-bromo-1-(4-toluene­sulfon­yl)-1*H*-pyrrol-2-yl]-1,3-di­nitro­butan-2-yl}-1-(4-toluene­sulfon­yl)-1*H*-pyrrole, another ostensible by-product in the synthesis of *geminal*-dimethyl hydro­dipyrrins

**DOI:** 10.1107/S2056989023004644

**Published:** 2023-06-02

**Authors:** Harry C. Sample, Brendan Twamley, Mathias O. Senge

**Affiliations:** a School of Chemistry, Chair of Organic Chemistry, Trinity Biomedical Sciences Institute, Trinity College Dublin, 152-160 Pearse St., D02 R590, Dublin, Ireland; b School of Chemistry, Trinity College Dublin, College Green, Dublin 2, Ireland; Katholieke Universiteit Leuven, Belgium

**Keywords:** crystal structure, enanti­omer, resolution, pyrrole, by-product

## Abstract

The crystal structure of (*R*,*R*)-4-bromo-2-{4-[4-bromo-1-(4-toluene­sulfon­yl)-1*H*-pyrrol-2-yl]-1,3-di­nitro­butan-2-yl}-1-(4-toluene­sulfon­yl)-1*H*-pyrrole (**1**, C_26_H_24_Br_2_N_4_O_8_S_2_) is presented. The title compound was isolated in suitable yield as a by-product in our synthesis of *geminal*-dimethyl hydro­dipyrrins. We observe an unforeseen enanti­omeric resolution both in the bulk sample and the crystal of **1**, with distinct C—H⋯O (C_meth­yl_—H⋯O_nitro_, C*sp* ^3^—H⋯O_sulfon­yl_) inter­actions observed in the enanti­omers present, along with other inter­actions, namely C_5-pyrrol­yl_—H⋯O_sulfon­yl_, forming a polymer along the crystallographic *c*-axis direction. Whilst pyrrolic fragments are well documented in the literature, little data is found surrounding the 1,3-di­nitro­butane scaffold.

## Abstract 2

The crystal structure of a by-product in the synthesis of *geminal*-dimethyl hydro­dipyrrins is reported; (*R*,*R*)-4-bromo-2-{4-[4-bromo-1-(4-toluene­sulfon­yl)-1*H*-pyrrol-2-yl]-1,3-di­nitro­butan-2-yl}-1-(4-toluene­sulfon­yl)-1*H*-pyrrole (**1**, C_26_H_24_N_4_O_8_S_2_Br_2_). Generated through a nitro­nate-mediated dimerization, this compound presents unforeseen enanti­omeric resolution, something previously not noted in its singular prior report. This crystal adds to the ever-growing library of by-products arising from these syntheses.

## 1. Chemical context

*geminal*-Dimethyl hydro­porphyrins were first made a reality *via* the *de novo* syntheses of (±)-bonellin presented in the 1980s and 1990s (Dutton *et al.*, 1983 ▸; Montforts & Schwartz, 1991 ▸). However, for modern oxidation-resistant chlorins, we look to the Lindsey group (Lindsey, 2015 ▸). Beginning at the turn of the century (Strachan *et al.*, 2000 ▸), their extension of Battersby’s thermal route has become the go-to synthesis for oxidation-resistant hydro­porphyrins. Since its inception there have been multiple refinements (Ptaszek *et al.*, 2005 ▸; Laha *et al.*, 2006 ▸; Krayer *et al.*, 2009 ▸). Subsequently, this synthesis has found applications in understanding the electronics of the chlorin macrocycle (Mass *et al.*, 2009 ▸), the generation of *E*-ring-functionalized hydro­porphyrins (Ptaszek *et al.*, 2010 ▸), the generation of hydro­porphyrin dimers and arrays (Meares *et al.*, 2015 ▸), and taking steps towards generating *N*-confused oxidation-resistant hydro­porphyrins (Xiong *et al.*, 2019 ▸).

Noted only once previously is the formation of a by-product, **1** (Krayer *et al.*, 2009 ▸). Through our own ventures into the world of hydro­porphyrins (Melissari *et al.*, 2020 ▸; Kingsbury *et al.*, 2021 ▸), we have in one instance generated a suitable amount of dimeric by-product **1**, and single crystals therefrom. The crystal structure of this elusive by-product, obtained in the synthesis of *geminal*-dimethyl hydro­dipyrrins and hydro­porphyrins, is described in this work. The structure presented in this work adds to an ever-increasing library of by-products from this field, which includes tricyclic undecane (CSD refcode CAJVUF; Taniguchi *et al.*, 2001 ▸) and di­hydro­oxazine (BESZEI; Tran *et al.*, 2022 ▸). 
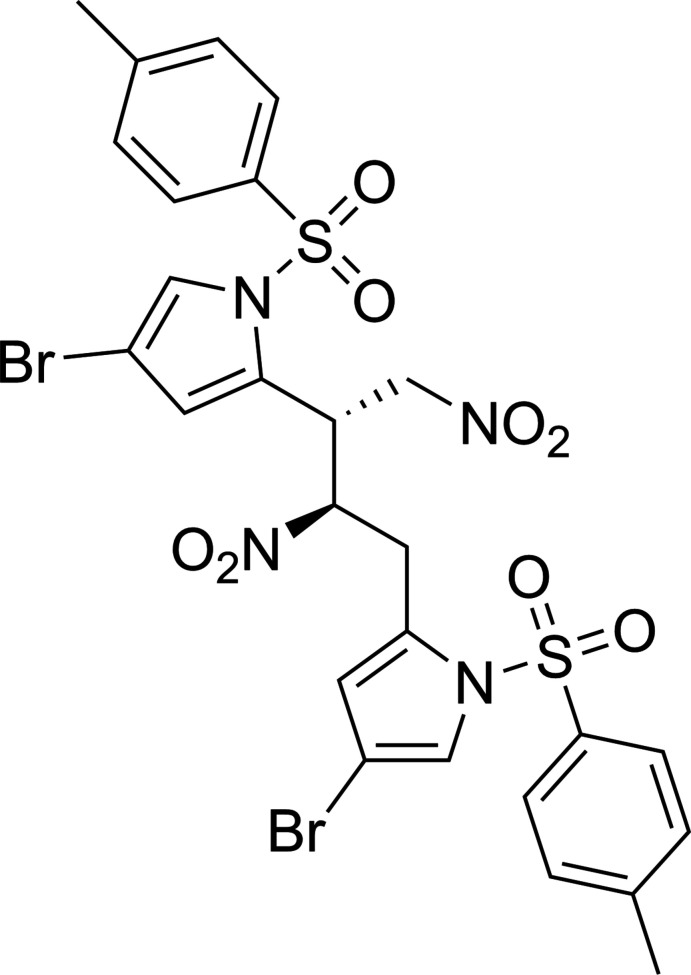


## 2. Structural commentary

**Figure 1:**
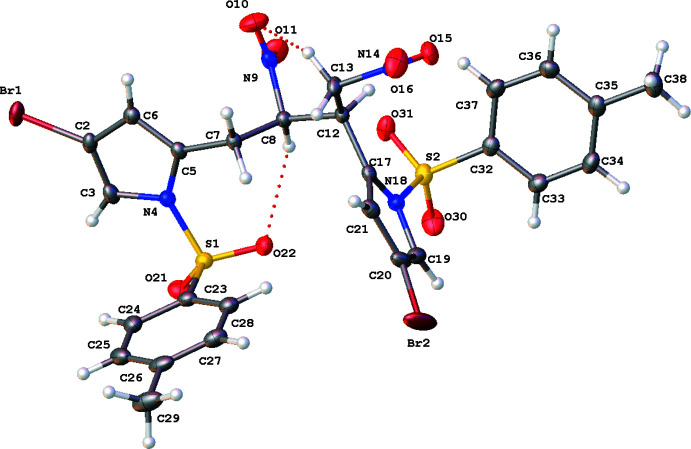
Mol­ecular structure of **1**. Displacement ellipsoids (non-H) are drawn at the 50% probability level, with H atoms presented as spheres of fixed radius (0.2 Å). Dotted lines indicate intra­molecular hydrogen bonding. Generated in *OLEX2* (Dolomanov *et al.*, 2009 ▸).

**Figure 2:**
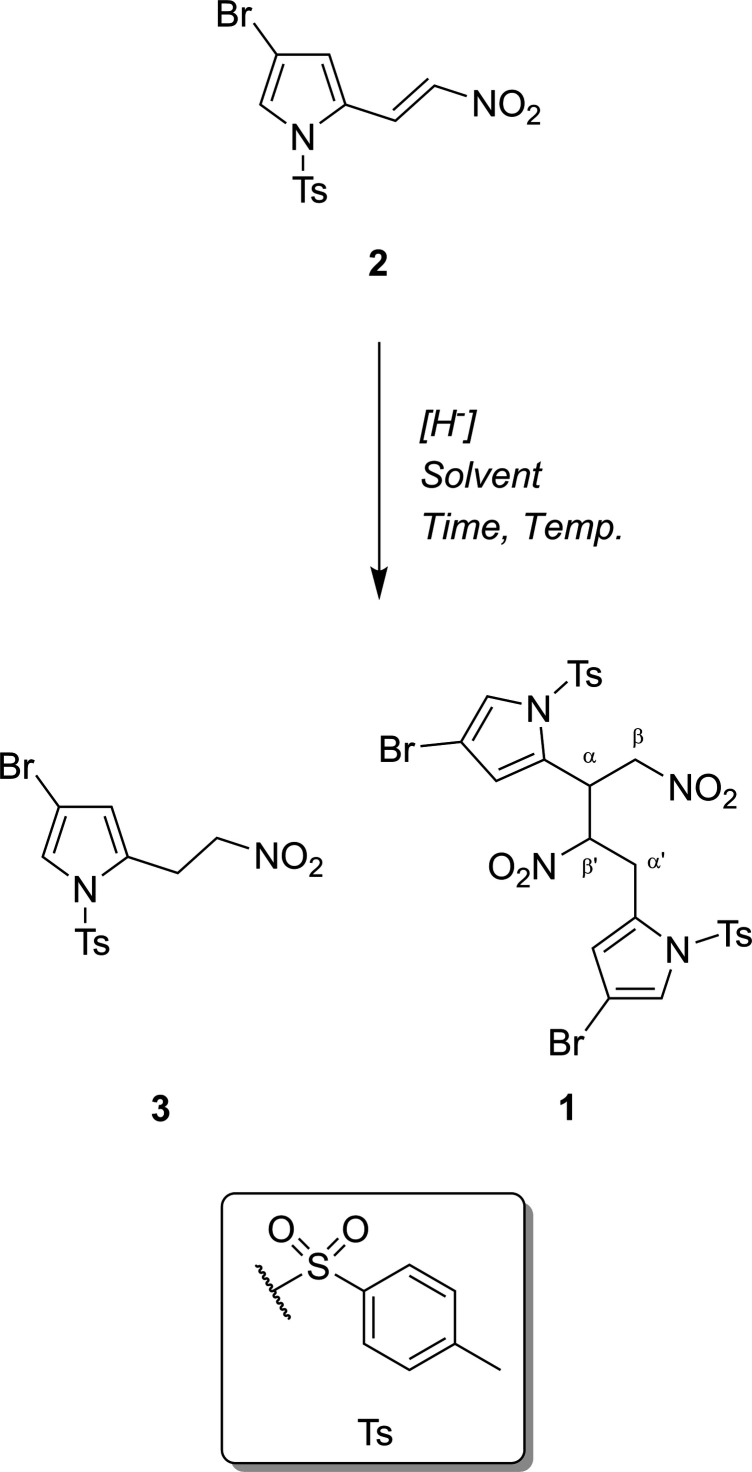
Synthesis of dimeric by-product **1** through the reduction of **2** to yield **3**. Reagents are non-specific given the number of differing procedures in the literature. α and β labels added to heighten the disymmetry of **1**.

The title compound **1** presents an asymmetric unit of one mol­ecule of the title compound with no solvate. Compound **1** was found to crystallize in the ortho­rhom­bic system (*Pbca*, *Z* = 8). Although a chiral compound, this is a racemate and the asymmetric unit is shown in Fig. 1 ▸ as (*R*,*R*)-stereochemistry. In ^1^H NMR spectroscopy, along with the respective 2D NMR with analyses undertaken of the same sample, we observe only one set of resonances for the aliphatic nitro­butane system (full ^1^H, ^13^C and ^1^H-^13^C HSQC NMR spectra are presented in the supporting information). The implication herein is that the sample presented contains the enanti­omers (*R*,*R*) and (*S*,*S*) only, with no other diastereomers present; see Fig. 2 ▸ for the synthetic pathway.

Both pyrrole rings are essentially planar, with RMSD values of 0.009 Å in both instances, and exhibit bond distances comparable with previous data (Kingsbury *et al.*, 2021 ▸). Both tosyl groups also exhibit the same conformation, *i.e*. with the *p*-tolyl ring coming out of the plane of the pyrrole ring, when viewing the respective pyrrole ring face on, as shown in Fig. 1 ▸, with N—S—C angles of 104.36 (9) and 105.26 (10)°, with the larger angle arising in the motif exhibiting an intra­molecular C*sp* ^3^–H⋯O_sulfon­yl_ inter­action (see Table 1 ▸). Despite the hydrogen-bonding inter­actions present, the O=S=O angle changes minimally 120.34 (10)°, in comparison to 120.86 (11)° for the non-inter­acting tosyl moiety. The dihedral angle between the pyrrole rings is 72.00 (12)°. The bond distances are within normal ranges (Groom *et al.*, 2016 ▸).

Lacking any protic donor or more traditional strong supra­molecular inter­actions, this structure is dominated by weaker C—H⋯O inter­actions; see Table 1 ▸. There are several intra­molecular C—H⋯O inter­actions. In the case of the bifurcated C8⋯O22_sulfon­yl_ and C8⋯O31_sulfon­yl_ inter­actions of 3.071 (3) and 3.072 (3) Å, we observe seven-membered ring formation. In another bifurcated intra­molecular inter­action, C12⋯O15_nitro_ and C12⋯O31_sulfon­yl_, 2.719 (2) and 2.913 (3) Å differing sized rings are formed, with the inter­action between methine and nitro motifs yielding a five-membered ring, and a six-membered ring between the methine and sulfonyl motifs. With C13*sp* ^3^⋯O10_nitro_ at 3.038 (3)Å, we observe one of the two nitro groups forming a six-membered ring with an opposing nitro­methyl motif.

We have no mechanistic evidence to rationalize the generation of **1**, be it through a non-stereoselective nitro­nate addition followed by kinetic precipitation to yield **1**, or simply through the impossibility of the formation of (*R*,*S*)-**1** or (*S*,*R*)-**1** as a direct result of steric inter­actions between two 1,2,4-tris­ubstituted pyrrolic motifs.

## 3. Supra­molecular features

**Figure 3:**
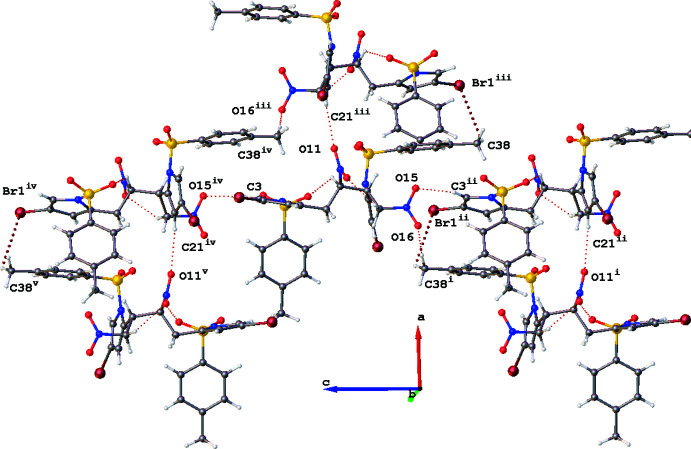
Inter­molecular inter­actions shown normal to the *c* axis. Only the atoms involved in these inter­actions are labelled. Generated in *OLEX2* (Dolomanov *et al.*, 2009 ▸). Symmetry codes: (i) − 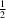 + *x*, *y*, 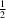 − *z*; (ii) *x*, 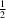 − *y*, − 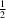 + *z*; (iii) 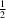 + *x*, 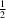 − *y*, 1 − *z*; (iv) *x*, 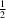 − *y*, 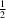 + *z*; (v) − 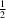 + *x*, *y*, 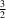 − *z*.

Regarding inter­molecular inter­actions, there are several C—H⋯O synthons present involving the nitro motifs. The first is seen with the opposite oxygen to the intra­molecular synthon described above, with the bromo­pyrrole linking to the adjacent nitro group, C21⋯O11^ii^, 3.459 (3) Å. The second involves the other nitro­methyl motif which exhibits a C3⋯O15^i^ inter­action of 3.194 (3) Å with an adjacent mol­ecule of the title compound arising from the 5-pyrrolyl position. The other nitro oxygen is involved with the methyl group on the tosyl phenyl ring with C38_meth­yl_⋯O16^iv^, 3.478 (3) Å and this also brings the methyl group into alignment with a neighbouring bromine, C38⋯Br1^v^, 3.639 (3) Å. These two inter­actions propagate along the crystallographic *c*-axis direction, which is shown in Fig. 3 ▸, forming loosely associated sheets. These sheets are weakly connected by C27_tos­yl_⋯O10_nitro_^iii^, 3.413 (3) Å.

## 4. Database survey

A search in the Cambridge Structural Database (CSD, Version 5.43, update of November 2022; Groom *et al.*, 2016 ▸) for the 4-bromo-2-(2-nitro­eth­yl)-1λ^2^-pyrrole subunit reveals only a few hits: HULBIA (Krayer *et al.*, 2009 ▸), OXIKAK (Chung *et al.*, 2021 ▸) and UNOYOO (Kingsbury *et al.*, 2021 ▸). In each of these compounds, the pyrrole is protected by a *p*-tosyl­ate group, as seen in **1**, and bond lengths are similar within the 2-(2-nitro­eth­yl)pyrrole moiety. Widening the parameters to the non-halogenated 2-(2-nitro­eth­yl)-1λ^2^-pyrrole subunit does reveal several more structures, ranging from asymmetric Friedel–Crafts alkyl­ation products as seen in KETBER (Stadler *et al.*, 2006 ▸) and DADYIS (Arai *et al.*, 2011 ▸), precursors in the synthesis of bacteriochlorins MIQHOL, MIQHUR (Jiang *et al.*, 2014 ▸), OXIJUD (Chung *et al.*, 2021 ▸) and CAXLEW (Jing *et al.*, 2022 ▸) and building blocks for the synthesis of β-substituted chlorins (QEZCED; Balasubramanian *et al.*, 2000 ▸).

A search encompassing the fragment 2-methyl-1,3-di­nitro­butane was undertaken and a large number of structures returned, many containing nitro-adamantyl and nitro-cubane motifs (Zhang *et al.*, 2000 ▸). Other motifs presented revealed strained geometries, *e.g.*, 1,3-di­nitro­cyclo­butane motifs. There were very few results of suitable structural similarity, those being DISGIX (Singha Roy & Mukherjee, 2014 ▸) and WOFJUX (Rabong *et al.*, 2008 ▸). Across the series of metrics for these three structures, all values regarding the nitro­butane system are roughly within accordance to those presented herein. As noted *vide supra*, the pyrrolic fragments remain consistent with data previously reported (Kingsbury *et al.*, 2021 ▸).

## 5. Synthesis and crystallization

Compounds **2** and **3** were synthesized following the reported procedures (Krayer *et al.*, 2009 ▸). For **1**, crystals were generated *via* slow evaporation at room temperature of a saturated solution of **1** in CDCl_3_. We have previously described the crystallization of **2** (Kingsbury *et al.*, 2021 ▸) and currently no structure of **3** has been reported. Compound **1** was obtained in 10% yield from **2**, with yields for **3** we typically observe approx. 69%, close to those previously reported (Laha *et al.*, 2006 ▸).

^1^H NMR spectroscopic data matched previously reported compounds **2** and **3**. Whilst the isolation of compound **1** has been reported previously, no comment on its stereochemistry has been presented. Below, we present analytical data for (*R*,*R*)-**1**, and within the supporting information, we have attached the appropriate spectra, Figs. S1–S3. Furthermore, we also present the ^1^H NMR spectra of **2** and **3** overlayed with the ^1^H NMR spectra of (*R*,*R*)-**1** for completeness (Fig. S4).

Analytical data for (*R*,*R*)-**1**: ^1^H NMR (298 K, CDCl_3_, 600 MHz): *δ* = 7.77 (*d*, *J* = 8.3 Hz, 2H), 7.61 (*d*, *J* = 8.3 Hz, 2H), 7.42 (*s*, 1H), 7.38 (*d*, *J* = 8.3 Hz, 2H), 7.36 (*d*, *J* = 8.2 Hz, 2H), 7.30 (*d*, *J* = 1.6 Hz, 1H), 6.17 (*d*, *J* = 1.0 Hz, 1H), 5.99 (*s*, 1H), 5.29–5.32 (*m*, 1H), 4.93–4.96 (*m*, 1H), 4.77–4.80 (*m*, 1H), 4.44–4.47 (*m*, 1H), 3.27–3.30 (*m*, 1H), 3.07–3.12 (*m*, 1H), 2.45 (*s*, 3H), 2.44 (*s*, 3H) ppm; ^13^C{^1^H} NMR (298 K, CDCl_3_, 151 MHz): *δ* = 146.8, 146.2, 135.2, 134.6, 130.7 (2), 130.7 (0) 130.6, 128.0, 127.4, 127.0, 123.9, 122.8, 118.5, 117.2, 100.9 (5), 100.9 (3), 87.8, 74.2, 37.8, 27.9, 21.9, 21.8 ppm; HRMS (ESI^−^) *m*/*z* calculated for [C_26_H_24_N_4_O_8_S_2_Br_2_+Cl]^−^, [*M* + Cl]^−^: 776.9096, found: 776.9075; *R*_F_ = 0.70 (silica, CH_2_Cl_2_:C_6_H_14_, 3:1); m.p.: 493–496 K (dec.), lit. (Krayer *et al.*, 2009 ▸) 388–390 K.

## 6. Refinement

Crystal data, data collection and structure refinement details are summarized in Table 2 ▸. Hydrogen atoms were positioned geometrically and refined isotropically using a riding model with C—H = 0.93–0.98 Å and *U*_iso_(H) = 1.2–1.5*U*_eq_(C).

## Supplementary Material

Crystal structure: contains datablock(s) I, global. DOI: 10.1107/S2056989023004644/vm2284sup1.cif

Structure factors: contains datablock(s) I. DOI: 10.1107/S2056989023004644/vm2284Isup2.hkl

PDF Document containing 1H, 13C{1H}, and 1H-13-HSQC NMR Spectra of compound 1, and overlayed 1H NMR spectra of compounds 1, 2, and 3. DOI: 10.1107/S2056989023004644/vm2284sup3.pdf

Supporting information file. DOI: 10.1107/S2056989023004644/vm2284Isup4.cml

CCDC reference: 2265533

Additional supporting information:  crystallographic information; 3D view; checkCIF report

## Figures and Tables

**Table 1:** Hydrogen-bond geometry (Å, °)

*D*—H⋯*A*	*D*—H	H⋯*A*	*D*⋯*A*	*D*—H⋯*A*
C3—H3⋯O15^i^	0.95	2.29	3.194 (3)	158
C8—H8⋯O22	1.00	2.38	3.071 (3)	126
C8—H8⋯O31	1.00	2.57	3.072 (3)	111
C13—H13*A*⋯O10	0.99	2.31	3.038 (3)	130
C21—H21⋯O11^ii^	0.95	2.63	3.459 (3)	146
C27—H27⋯O10^iii^	0.95	2.75	3.413 (3)	128
C38—H38*B*⋯O16^iv^	0.98	2.51	3.478 (3)	170
C38—H38*C*⋯Br1^v^	0.98	3.33	3.639 (3)	100

Symmetry codes: (i) 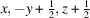 ; (ii) 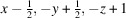 ; (iii) 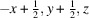 ; (iv) 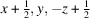 ; (v) 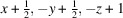 .

**Table 2:** Experimental details

Crystal data	
Chemical formula	C_26_H_24_Br_2_N_4_O_8_S_2_
*M*_r_	744.43
Crystal system, space group	Orthorhombic, *P* *b* *c* *a*
Temperature (K)	100
*a*, *b*, *c* (Å)	13.9764 (7), 17.8228 (9), 23.0590 (11)
*V* (Å^3^)	5744.0 (5)
*Z*	8
Radiation type	Cu *K*α
μ (mm^−1^)	5.43
Crystal size (mm)	0.41 × 0.14 × 0.13

Data collection	
Diffractometer	Bruker *APEX2* Kappa Duo
Absorption correction	Multi-scan (*SADABS*; Krause *et al.*, 2015 ▸)
*T*_min_, *T*_max_	0.429, 0.753
No. of measured, independent and observed [*I* > 2σ(*I*)] reflections	55114, 5407, 5392
*R*_int_	0.040
(sin θ/λ)_max_ (Å^−1^)	0.609

Refinement	
*R*[*F* ^2^ > 2σ(*F* ^2^)], *wR*(*F* ^2^), *S*	0.030, 0.080, 1.08
No. of reflections	5407
No. of parameters	381
H-atom treatment	H-atom parameters constrained
Δρ_max_, Δρ_min_ (e Å^−3^)	1.03, −1.27

Computer programs: *APEX3* (Bruker, 2017 ▸), *SAINT* (Bruker, 2018 ▸), *SHELXT* (Sheldrick, 2015*a* ▸), *SHELXL* (Sheldrick, 2015*b* ▸) and *OLEX2* (Dolomanov *et al.*, 2009 ▸).

## supplementary crystallographic information

### Crystal data

**Table:**

C_26_H_24_Br_2_N_4_O_8_S_2_	*D*_x_ = 1.722 Mg m^−^^3^
*M**_r_* = 744.43	Cu *K*α radiation, λ = 1.54178 Å
Orthorhombic, *P**b**c**a*	Cell parameters from 9744 reflections
*a* = 13.9764 (7) Å	θ = 3.7–69.7°
*b* = 17.8228 (9) Å	µ = 5.43 mm^−^^1^
*c* = 23.0590 (11) Å	*T* = 100 K
*V* = 5744.0 (5) Å^3^	Block, colourless
*Z* = 8	0.41 × 0.14 × 0.13 mm
*F*(000) = 2992	

### Data collection

**Table:**

Bruker APEX2 Kappa Duo diffractometer	5407 independent reflections
Radiation source: microfocus sealed X-ray tube, Incoatec Iµs	5392 reflections with *I* > 2σ(*I*)
Mirror optics monochromator	*R*_int_ = 0.040
Detector resolution: 8.33 pixels mm^-1^	θ_max_ = 69.9°, θ_min_ = 3.8°
ω and φ scans	*h* = −16→16
Absorption correction: multi-scan (SADABS; Krause *et al.*, 2015)	*k* = −20→21
*T*_min_ = 0.429, *T*_max_ = 0.753	*l* = −26→27
55114 measured reflections	

### Refinement

**Table:**

Refinement on *F*^2^	Primary atom site location: dual
Least-squares matrix: full	Hydrogen site location: inferred from neighbouring sites
*R*[*F*^2^ > 2σ(*F*^2^)] = 0.030	H-atom parameters constrained
*wR*(*F*^2^) = 0.080	*w* = 1/[σ^2^(*F*_o_^2^) + (0.0376*P*)^2^ + 8.4555*P*] where *P* = (*F*_o_^2^ + 2*F*_c_^2^)/3
*S* = 1.08	(Δ/σ)_max_ = 0.002
5407 reflections	Δρ_max_ = 1.03 e Å^−^^3^
381 parameters	Δρ_min_ = −1.26 e Å^−^^3^
0 restraints	

### Special details

**Table:**

Geometry. All esds (except the esd in the dihedral angle between two l.s. planes) are estimated using the full covariance matrix. The cell esds are taken into account individually in the estimation of esds in distances, angles and torsion angles; correlations between esds in cell parameters are only used when they are defined by crystal symmetry. An approximate (isotropic) treatment of cell esds is used for estimating esds involving l.s. planes.

### Fractional atomic coordinates and isotropic or equivalent isotropic displacement parameters (Å^2^)

**Table:**

	*x*	*y*	*z*	*U*_iso_*/*U*_eq_	
Br1	0.38552 (2)	0.11603 (2)	0.78537 (2)	0.02394 (8)	
Br2	0.26600 (2)	0.50453 (2)	0.40962 (2)	0.04351 (10)	
C2	0.38553 (15)	0.17833 (13)	0.71975 (9)	0.0179 (4)	
C3	0.40706 (16)	0.25232 (13)	0.72050 (9)	0.0196 (4)	
H3	0.422447	0.281594	0.753638	0.024*	
C5	0.37376 (14)	0.21731 (12)	0.62743 (9)	0.0155 (4)	
C6	0.36492 (15)	0.15580 (12)	0.66188 (9)	0.0167 (4)	
H6	0.348050	0.106685	0.649553	0.020*	
C7	0.35869 (14)	0.22272 (12)	0.56344 (9)	0.0159 (4)	
H7A	0.322995	0.269432	0.554982	0.019*	
H7B	0.318434	0.180014	0.550858	0.019*	
C8	0.45083 (15)	0.22258 (11)	0.52779 (9)	0.0157 (4)	
H8	0.493485	0.263372	0.542559	0.019*	
C12	0.43434 (15)	0.23502 (11)	0.46193 (8)	0.0155 (4)	
H12	0.493947	0.219779	0.441034	0.019*	
C13	0.35197 (17)	0.18725 (12)	0.43891 (9)	0.0203 (4)	
H13A	0.356118	0.136153	0.455558	0.024*	
H13B	0.290293	0.209552	0.451093	0.024*	
C17	0.41664 (15)	0.31683 (12)	0.44962 (8)	0.0154 (4)	
C19	0.44913 (17)	0.44091 (12)	0.43708 (9)	0.0228 (5)	
H19	0.482321	0.487203	0.433591	0.027*	
C20	0.35401 (18)	0.42997 (13)	0.43040 (9)	0.0236 (5)	
C21	0.33245 (16)	0.35280 (13)	0.43881 (9)	0.0196 (4)	
H21	0.270801	0.330445	0.437198	0.024*	
C23	0.28460 (16)	0.39795 (12)	0.64711 (10)	0.0189 (4)	
C24	0.23767 (17)	0.40596 (12)	0.70006 (10)	0.0233 (5)	
H24	0.269146	0.393877	0.735365	0.028*	
C25	0.14427 (18)	0.43187 (13)	0.70024 (11)	0.0274 (5)	
H25	0.111262	0.437031	0.736034	0.033*	
C26	0.09807 (17)	0.45044 (13)	0.64881 (12)	0.0275 (5)	
C27	0.14605 (17)	0.44162 (13)	0.59641 (11)	0.0254 (5)	
H27	0.114396	0.453603	0.561148	0.031*	
C28	0.23991 (17)	0.41545 (12)	0.59498 (10)	0.0212 (5)	
H28	0.272671	0.409680	0.559160	0.025*	
C29	−0.00249 (19)	0.48175 (15)	0.65026 (15)	0.0391 (7)	
H29A	−0.000681	0.533976	0.663503	0.059*	
H29B	−0.041680	0.451961	0.676941	0.059*	
H29C	−0.030263	0.479592	0.611271	0.059*	
C32	0.62371 (14)	0.34767 (12)	0.36715 (9)	0.0180 (4)	
C33	0.63329 (16)	0.41207 (13)	0.33356 (10)	0.0215 (4)	
H33	0.634478	0.460162	0.351316	0.026*	
C34	0.64106 (16)	0.40521 (13)	0.27395 (10)	0.0222 (5)	
H34	0.648336	0.448962	0.250835	0.027*	
C35	0.63835 (15)	0.33521 (13)	0.24746 (10)	0.0212 (4)	
C36	0.63108 (16)	0.27134 (13)	0.28224 (10)	0.0217 (5)	
H36	0.631052	0.223194	0.264561	0.026*	
C37	0.62390 (15)	0.27679 (13)	0.34204 (10)	0.0206 (4)	
H37	0.619199	0.233004	0.365358	0.025*	
C38	0.64096 (18)	0.32774 (15)	0.18235 (10)	0.0280 (5)	
H38A	0.576947	0.315054	0.168054	0.042*	
H38B	0.685970	0.287978	0.171515	0.042*	
H38C	0.661671	0.375340	0.165169	0.042*	
N4	0.40231 (13)	0.27742 (10)	0.66314 (8)	0.0167 (4)	
N9	0.50181 (14)	0.14805 (11)	0.53523 (7)	0.0202 (4)	
N14	0.35545 (15)	0.18255 (10)	0.37393 (8)	0.0222 (4)	
N18	0.48917 (13)	0.37130 (10)	0.45008 (8)	0.0176 (4)	
O10	0.45646 (14)	0.09033 (10)	0.52684 (9)	0.0353 (4)	
O11	0.58591 (13)	0.14916 (11)	0.54810 (9)	0.0350 (4)	
O15	0.43320 (13)	0.18728 (10)	0.34992 (7)	0.0294 (4)	
O16	0.28002 (15)	0.17100 (12)	0.34869 (8)	0.0381 (5)	
O21	0.45337 (12)	0.40320 (9)	0.69459 (7)	0.0260 (4)	
O22	0.44181 (11)	0.37517 (9)	0.58944 (7)	0.0218 (3)	
O30	0.65028 (13)	0.42732 (11)	0.45989 (8)	0.0321 (4)	
O31	0.63020 (11)	0.28973 (10)	0.47091 (7)	0.0252 (4)	
S1	0.40504 (4)	0.36902 (3)	0.64696 (2)	0.01761 (12)	
S2	0.60804 (4)	0.35847 (3)	0.44193 (2)	0.02007 (12)	

### Atomic displacement parameters (Å^2^)

**Table:**

	*U*^11^	*U*^22^	*U*^33^	*U*^12^	*U*^13^	*U*^23^
Br1	0.02037 (13)	0.03358 (15)	0.01786 (13)	0.00193 (9)	0.00022 (8)	0.01063 (9)
Br2	0.0594 (2)	0.03287 (16)	0.03827 (17)	0.02900 (14)	−0.02077 (14)	−0.01200 (11)
C2	0.0139 (10)	0.0252 (11)	0.0147 (10)	0.0040 (8)	0.0017 (7)	0.0048 (8)
C3	0.0190 (10)	0.0276 (11)	0.0124 (10)	0.0041 (9)	−0.0011 (8)	−0.0009 (8)
C5	0.0131 (9)	0.0194 (10)	0.0141 (10)	0.0017 (8)	−0.0002 (7)	−0.0009 (8)
C6	0.0134 (9)	0.0193 (10)	0.0173 (10)	0.0016 (8)	0.0013 (8)	0.0010 (8)
C7	0.0141 (9)	0.0207 (10)	0.0130 (10)	0.0005 (8)	0.0003 (8)	0.0000 (8)
C8	0.0165 (10)	0.0167 (10)	0.0139 (10)	0.0008 (8)	0.0000 (8)	0.0001 (7)
C12	0.0171 (10)	0.0169 (10)	0.0124 (9)	−0.0005 (8)	0.0016 (8)	−0.0001 (7)
C13	0.0279 (12)	0.0212 (10)	0.0120 (10)	−0.0060 (9)	−0.0003 (8)	−0.0001 (8)
C17	0.0166 (10)	0.0187 (10)	0.0108 (9)	−0.0011 (8)	−0.0002 (7)	−0.0011 (7)
C19	0.0324 (13)	0.0168 (10)	0.0191 (10)	0.0023 (9)	0.0012 (9)	−0.0010 (8)
C20	0.0321 (12)	0.0217 (11)	0.0169 (10)	0.0115 (9)	−0.0039 (9)	−0.0035 (8)
C21	0.0183 (10)	0.0247 (11)	0.0159 (10)	0.0025 (9)	−0.0016 (8)	−0.0031 (8)
C23	0.0193 (10)	0.0131 (9)	0.0243 (11)	0.0004 (8)	0.0020 (8)	−0.0004 (8)
C24	0.0293 (12)	0.0167 (10)	0.0239 (11)	−0.0014 (9)	0.0048 (9)	0.0010 (9)
C25	0.0296 (13)	0.0176 (11)	0.0349 (13)	−0.0002 (9)	0.0128 (11)	−0.0008 (9)
C26	0.0221 (12)	0.0135 (10)	0.0469 (15)	−0.0007 (9)	0.0050 (10)	0.0020 (10)
C27	0.0240 (12)	0.0179 (11)	0.0344 (13)	0.0001 (9)	−0.0042 (10)	0.0030 (9)
C28	0.0238 (11)	0.0158 (10)	0.0239 (11)	−0.0004 (9)	0.0005 (9)	0.0000 (8)
C29	0.0223 (12)	0.0221 (12)	0.073 (2)	0.0019 (10)	0.0084 (13)	0.0039 (13)
C32	0.0116 (9)	0.0224 (11)	0.0201 (10)	0.0001 (8)	0.0001 (8)	0.0029 (8)
C33	0.0180 (10)	0.0189 (11)	0.0276 (12)	−0.0022 (8)	0.0011 (9)	0.0013 (9)
C34	0.0186 (10)	0.0237 (11)	0.0242 (11)	−0.0020 (9)	0.0013 (9)	0.0080 (9)
C35	0.0136 (10)	0.0277 (11)	0.0223 (11)	0.0022 (8)	0.0019 (8)	0.0035 (9)
C36	0.0177 (10)	0.0223 (11)	0.0249 (11)	0.0039 (9)	0.0019 (8)	−0.0013 (9)
C37	0.0160 (10)	0.0197 (11)	0.0263 (11)	0.0026 (8)	0.0012 (8)	0.0054 (9)
C38	0.0243 (12)	0.0385 (14)	0.0213 (11)	0.0000 (10)	0.0025 (9)	0.0036 (10)
N4	0.0180 (8)	0.0172 (9)	0.0148 (8)	0.0016 (7)	−0.0004 (7)	−0.0003 (7)
N9	0.0227 (10)	0.0239 (10)	0.0138 (8)	0.0052 (8)	0.0017 (7)	0.0013 (7)
N14	0.0351 (11)	0.0157 (9)	0.0158 (9)	−0.0048 (8)	−0.0053 (8)	0.0005 (7)
N18	0.0180 (9)	0.0171 (8)	0.0178 (9)	−0.0001 (7)	0.0020 (7)	−0.0008 (7)
O10	0.0391 (10)	0.0188 (8)	0.0481 (11)	0.0008 (7)	−0.0077 (9)	−0.0008 (8)
O11	0.0229 (9)	0.0383 (10)	0.0439 (11)	0.0089 (8)	−0.0064 (8)	0.0093 (8)
O15	0.0381 (10)	0.0335 (9)	0.0167 (8)	−0.0003 (8)	0.0045 (7)	−0.0013 (7)
O16	0.0446 (11)	0.0455 (11)	0.0243 (9)	−0.0158 (9)	−0.0131 (8)	0.0018 (8)
O21	0.0262 (8)	0.0235 (8)	0.0285 (9)	−0.0030 (7)	−0.0046 (7)	−0.0069 (7)
O22	0.0225 (8)	0.0206 (8)	0.0224 (8)	−0.0010 (6)	0.0049 (6)	0.0025 (6)
O30	0.0302 (9)	0.0379 (10)	0.0282 (9)	−0.0165 (8)	−0.0019 (7)	−0.0045 (7)
O31	0.0155 (7)	0.0363 (9)	0.0238 (8)	0.0023 (7)	−0.0002 (6)	0.0092 (7)
S1	0.0178 (2)	0.0160 (2)	0.0190 (3)	−0.00104 (19)	−0.00008 (19)	−0.00084 (19)
S2	0.0150 (3)	0.0262 (3)	0.0189 (3)	−0.0046 (2)	−0.00105 (19)	0.0010 (2)

### Geometric parameters (Å, º)

**Table:**

Br1—C2	1.877 (2)	C25—C26	1.390 (4)
Br2—C20	1.873 (2)	C26—C27	1.391 (4)
C2—C3	1.353 (3)	C26—C29	1.513 (3)
C2—C6	1.423 (3)	C27—H27	0.9500
C3—H3	0.9500	C27—C28	1.393 (3)
C3—N4	1.398 (3)	C28—H28	0.9500
C5—C6	1.359 (3)	C29—H29A	0.9800
C5—C7	1.494 (3)	C29—H29B	0.9800
C5—N4	1.409 (3)	C29—H29C	0.9800
C6—H6	0.9500	C32—C33	1.391 (3)
C7—H7A	0.9900	C32—C37	1.390 (3)
C7—H7B	0.9900	C32—S2	1.749 (2)
C7—C8	1.528 (3)	C33—H33	0.9500
C8—H8	1.0000	C33—C34	1.384 (3)
C8—C12	1.552 (3)	C34—H34	0.9500
C8—N9	1.517 (3)	C34—C35	1.390 (3)
C12—H12	1.0000	C35—C36	1.396 (3)
C12—C13	1.527 (3)	C35—C38	1.508 (3)
C12—C17	1.506 (3)	C36—H36	0.9500
C13—H13A	0.9900	C36—C37	1.386 (3)
C13—H13B	0.9900	C37—H37	0.9500
C13—N14	1.501 (3)	C38—H38A	0.9800
C17—C21	1.363 (3)	C38—H38B	0.9800
C17—N18	1.404 (3)	C38—H38C	0.9800
C19—H19	0.9500	N4—S1	1.6752 (19)
C19—C20	1.353 (4)	N9—O10	1.224 (3)
C19—N18	1.393 (3)	N9—O11	1.212 (3)
C20—C21	1.421 (3)	N14—O15	1.222 (3)
C21—H21	0.9500	N14—O16	1.222 (3)
C23—C24	1.393 (3)	N18—S2	1.6875 (19)
C23—C28	1.390 (3)	O21—S1	1.4260 (17)
C23—S1	1.760 (2)	O22—S1	1.4268 (16)
C24—H24	0.9500	O30—S2	1.4233 (18)
C24—C25	1.385 (4)	O31—S2	1.4295 (17)
C25—H25	0.9500		
			
C3—C2—Br1	124.50 (17)	C26—C27—H27	119.6
C3—C2—C6	109.40 (19)	C26—C27—C28	120.8 (2)
C6—C2—Br1	126.09 (17)	C28—C27—H27	119.6
C2—C3—H3	126.6	C23—C28—C27	118.6 (2)
C2—C3—N4	106.82 (19)	C23—C28—H28	120.7
N4—C3—H3	126.6	C27—C28—H28	120.7
C6—C5—C7	128.06 (19)	C26—C29—H29A	109.5
C6—C5—N4	107.30 (18)	C26—C29—H29B	109.5
N4—C5—C7	124.63 (18)	C26—C29—H29C	109.5
C2—C6—H6	126.2	H29A—C29—H29B	109.5
C5—C6—C2	107.57 (19)	H29A—C29—H29C	109.5
C5—C6—H6	126.2	H29B—C29—H29C	109.5
C5—C7—H7A	108.7	C33—C32—S2	118.05 (17)
C5—C7—H7B	108.7	C37—C32—C33	121.2 (2)
C5—C7—C8	114.37 (17)	C37—C32—S2	120.75 (17)
H7A—C7—H7B	107.6	C32—C33—H33	120.4
C8—C7—H7A	108.7	C34—C33—C32	119.2 (2)
C8—C7—H7B	108.7	C34—C33—H33	120.4
C7—C8—H8	108.5	C33—C34—H34	119.5
C7—C8—C12	113.65 (17)	C33—C34—C35	120.9 (2)
C12—C8—H8	108.5	C35—C34—H34	119.5
N9—C8—C7	109.66 (16)	C34—C35—C36	118.8 (2)
N9—C8—H8	108.5	C34—C35—C38	121.1 (2)
N9—C8—C12	107.79 (16)	C36—C35—C38	120.1 (2)
C8—C12—H12	108.0	C35—C36—H36	119.3
C13—C12—C8	111.87 (17)	C37—C36—C35	121.3 (2)
C13—C12—H12	108.0	C37—C36—H36	119.3
C17—C12—C8	110.31 (16)	C32—C37—H37	120.7
C17—C12—H12	108.0	C36—C37—C32	118.6 (2)
C17—C12—C13	110.51 (17)	C36—C37—H37	120.7
C12—C13—H13A	109.5	C35—C38—H38A	109.5
C12—C13—H13B	109.5	C35—C38—H38B	109.5
H13A—C13—H13B	108.1	C35—C38—H38C	109.5
N14—C13—C12	110.71 (17)	H38A—C38—H38B	109.5
N14—C13—H13A	109.5	H38A—C38—H38C	109.5
N14—C13—H13B	109.5	H38B—C38—H38C	109.5
C21—C17—C12	129.2 (2)	C3—N4—C5	108.85 (17)
C21—C17—N18	107.43 (18)	C3—N4—S1	121.44 (15)
N18—C17—C12	123.34 (18)	C5—N4—S1	128.12 (15)
C20—C19—H19	126.5	O10—N9—C8	118.35 (18)
C20—C19—N18	106.9 (2)	O11—N9—C8	117.95 (19)
N18—C19—H19	126.5	O11—N9—O10	123.7 (2)
C19—C20—Br2	124.92 (18)	O15—N14—C13	118.49 (18)
C19—C20—C21	109.4 (2)	O16—N14—C13	117.18 (19)
C21—C20—Br2	125.61 (18)	O16—N14—O15	124.26 (19)
C17—C21—C20	107.3 (2)	C17—N18—S2	128.06 (15)
C17—C21—H21	126.4	C19—N18—C17	108.91 (18)
C20—C21—H21	126.4	C19—N18—S2	119.48 (16)
C24—C23—S1	118.80 (18)	N4—S1—C23	105.26 (10)
C28—C23—C24	121.5 (2)	O21—S1—C23	109.05 (10)
C28—C23—S1	119.58 (17)	O21—S1—N4	104.80 (10)
C23—C24—H24	120.6	O21—S1—O22	120.83 (10)
C25—C24—C23	118.7 (2)	O22—S1—C23	108.89 (10)
C25—C24—H24	120.6	O22—S1—N4	106.87 (9)
C24—C25—H25	119.5	N18—S2—C32	104.36 (9)
C24—C25—C26	121.0 (2)	O30—S2—C32	109.26 (11)
C26—C25—H25	119.5	O30—S2—N18	105.02 (10)
C25—C26—C27	119.4 (2)	O30—S2—O31	120.86 (11)
C25—C26—C29	120.0 (2)	O31—S2—C32	109.84 (10)
C27—C26—C29	120.6 (2)	O31—S2—N18	106.11 (9)
			
Br1—C2—C3—N4	177.87 (14)	C19—N18—S2—O31	166.71 (16)
Br1—C2—C6—C5	−179.30 (15)	C20—C19—N18—C17	1.6 (2)
Br2—C20—C21—C17	176.01 (16)	C20—C19—N18—S2	162.05 (16)
C2—C3—N4—C5	2.2 (2)	C21—C17—N18—C19	−2.4 (2)
C2—C3—N4—S1	168.90 (15)	C21—C17—N18—S2	−160.62 (16)
C3—C2—C6—C5	−0.3 (2)	C23—C24—C25—C26	−0.7 (3)
C3—N4—S1—C23	−90.00 (18)	C24—C23—C28—C27	0.1 (3)
C3—N4—S1—O21	24.96 (19)	C24—C23—S1—N4	74.00 (19)
C3—N4—S1—O22	154.32 (17)	C24—C23—S1—O21	−38.0 (2)
C5—C7—C8—C12	−175.10 (17)	C24—C23—S1—O22	−171.71 (17)
C5—C7—C8—N9	64.2 (2)	C24—C25—C26—C27	1.1 (3)
C5—N4—S1—C23	73.98 (19)	C24—C25—C26—C29	−177.4 (2)
C5—N4—S1—O21	−171.06 (17)	C25—C26—C27—C28	−0.9 (3)
C5—N4—S1—O22	−41.7 (2)	C26—C27—C28—C23	0.3 (3)
C6—C2—C3—N4	−1.2 (2)	C28—C23—C24—C25	0.1 (3)
C6—C5—C7—C8	−100.9 (2)	C28—C23—S1—N4	−108.85 (18)
C6—C5—N4—C3	−2.4 (2)	C28—C23—S1—O21	139.16 (18)
C6—C5—N4—S1	−167.96 (15)	C28—C23—S1—O22	5.4 (2)
C7—C5—C6—C2	−179.46 (19)	C29—C26—C27—C28	177.6 (2)
C7—C5—N4—C3	178.67 (19)	C32—C33—C34—C35	0.7 (3)
C7—C5—N4—S1	13.1 (3)	C33—C32—C37—C36	−1.8 (3)
C7—C8—C12—C13	−45.4 (2)	C33—C32—S2—N18	85.18 (18)
C7—C8—C12—C17	78.0 (2)	C33—C32—S2—O30	−26.7 (2)
C7—C8—N9—O10	52.0 (2)	C33—C32—S2—O31	−161.43 (17)
C7—C8—N9—O11	−129.4 (2)	C33—C34—C35—C36	−2.3 (3)
C8—C12—C13—N14	−163.43 (17)	C33—C34—C35—C38	176.4 (2)
C8—C12—C17—C21	−102.3 (2)	C34—C35—C36—C37	1.8 (3)
C8—C12—C17—N18	74.7 (2)	C35—C36—C37—C32	0.2 (3)
C12—C8—N9—O10	−72.2 (2)	C37—C32—C33—C34	1.4 (3)
C12—C8—N9—O11	106.4 (2)	C37—C32—S2—N18	−93.07 (18)
C12—C13—N14—O15	28.9 (3)	C37—C32—S2—O30	155.04 (18)
C12—C13—N14—O16	−154.1 (2)	C37—C32—S2—O31	20.3 (2)
C12—C17—C21—C20	179.5 (2)	C38—C35—C36—C37	−176.9 (2)
C12—C17—N18—C19	−179.96 (18)	N4—C5—C6—C2	1.6 (2)
C12—C17—N18—S2	21.8 (3)	N4—C5—C7—C8	77.9 (2)
C13—C12—C17—C21	21.9 (3)	N9—C8—C12—C13	76.3 (2)
C13—C12—C17—N18	−161.05 (18)	N9—C8—C12—C17	−160.20 (17)
C17—C12—C13—N14	73.2 (2)	N18—C17—C21—C20	2.1 (2)
C17—N18—S2—C32	79.0 (2)	N18—C19—C20—Br2	−177.50 (15)
C17—N18—S2—O30	−166.11 (18)	N18—C19—C20—C21	−0.3 (2)
C17—N18—S2—O31	−37.0 (2)	S1—C23—C24—C25	177.21 (17)
C19—C20—C21—C17	−1.2 (3)	S1—C23—C28—C27	−176.98 (17)
C19—N18—S2—C32	−77.28 (18)	S2—C32—C33—C34	−176.85 (17)
C19—N18—S2—O30	37.63 (19)	S2—C32—C37—C36	176.36 (17)

### Hydrogen-bond geometry (Å, º)

**Table:**

*D*—H···*A*	*D*—H	H···*A*	*D*···*A*	*D*—H···*A*
C3—H3···O15^i^	0.95	2.29	3.194 (3)	158
C8—H8···O22	1.00	2.38	3.071 (3)	126
C8—H8···O31	1.00	2.57	3.072 (3)	111
C13—H13*A*···O10	0.99	2.31	3.038 (3)	130
C21—H21···O11^ii^	0.95	2.63	3.459 (3)	146
C27—H27···O10^iii^	0.95	2.75	3.413 (3)	128
C38—H38*B*···O16^iv^	0.98	2.51	3.478 (3)	170
C38—H38*C*···Br1^v^	0.98	3.33	3.639 (3)	100

Symmetry codes: (i) *x*, −*y*+1/2, *z*+1/2; (ii) *x*−1/2, −*y*+1/2, −*z*+1; (iii) −*x*+1/2, *y*+1/2, *z*; (iv) *x*+1/2, *y*, −*z*+1/2; (v) *x*+1/2, −*y*+1/2, −*z*+1.
