# Supplementary material for: Structure of (R,R)-4-bromo-2-{4-[4-bromo-1-(4-toluene­sulfon­yl)-1H-pyrrol-2-yl]-1,3-di­nitro­butan-2-yl}-1-(4-toluene­sulfon­yl)-1H-pyrrole, another ostensible by-product in the synthesis of geminal-dimethyl hydro­dipyrrins
Source: Acta Crystallogr E Crystallogr Commun. 2023 Jun 2;79(Pt 7):592–5. doi: 10.1107/S2056989023004644 (PMC10439434; doi:10.1107/S2056989023004644)
Supplement: Supplementary file 3 [file e-79-00592-sup3.pdf]

Structure of (*R*,*R*)-4-bromo-2-(4-(4-bromo-1-tosyl-1*H*-pyrrol-2-yl)-1,3-dinitrobutan-2-yl)-1-tosyl-1*H*-pyrrole, another ostensible by-product in the synthesis of *geminal*-dimethyl hydrodipyrrins.

Harry C. Sample,[a]* Brendan Twamley[b] and Mathias O. Senge[a]

[a]School of Chemistry, Chair of Organic Chemistry, Trinity Biomedical Sciences Institute, Trinity College Dublin, 152–160 Pearse St., D02 R590, Dublin, Ireland, and [b]School of Chemistry, Trinity College Dublin, College Green, Dublin 2, Ireland

Correspondence email: sampleh@tcd.ie

SUPPORTING INFORMATION

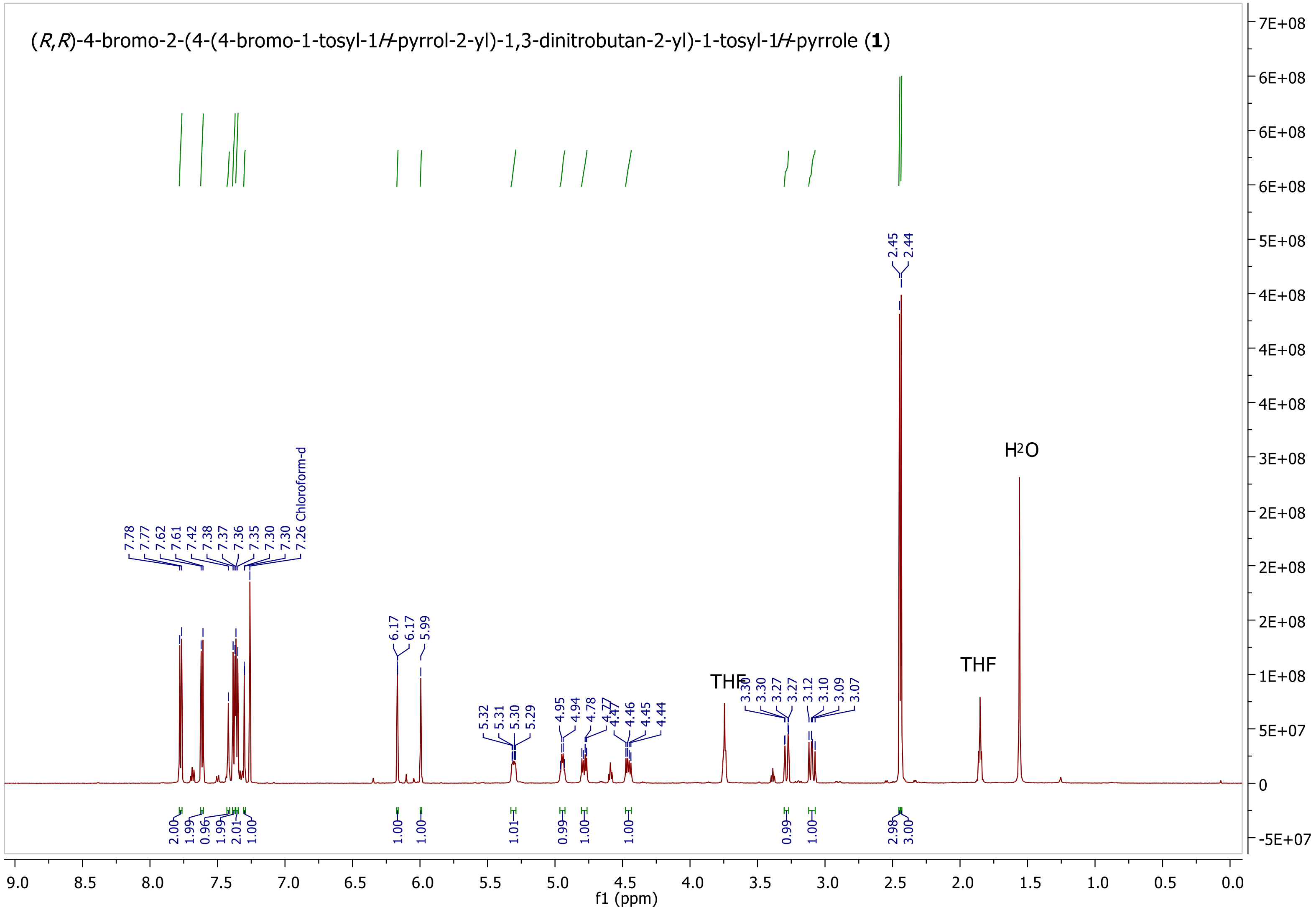

Figure S1. ^{1}H NMR spectra of (*R*,*R*)-4-bromo-2-(4-(4-bromo-1-tosyl-1*H*-pyrrol-2-yl)-1,3-dinitrobutan-2-yl)-1-tosyl-1*H*-pyrrole (**1**) ($CDCl_3$, 600 MHz, 298 K). Chemical shifts were determined with respect to the residual solvent peak. Residual THF and H_2O from the reaction mixture have been annotated.

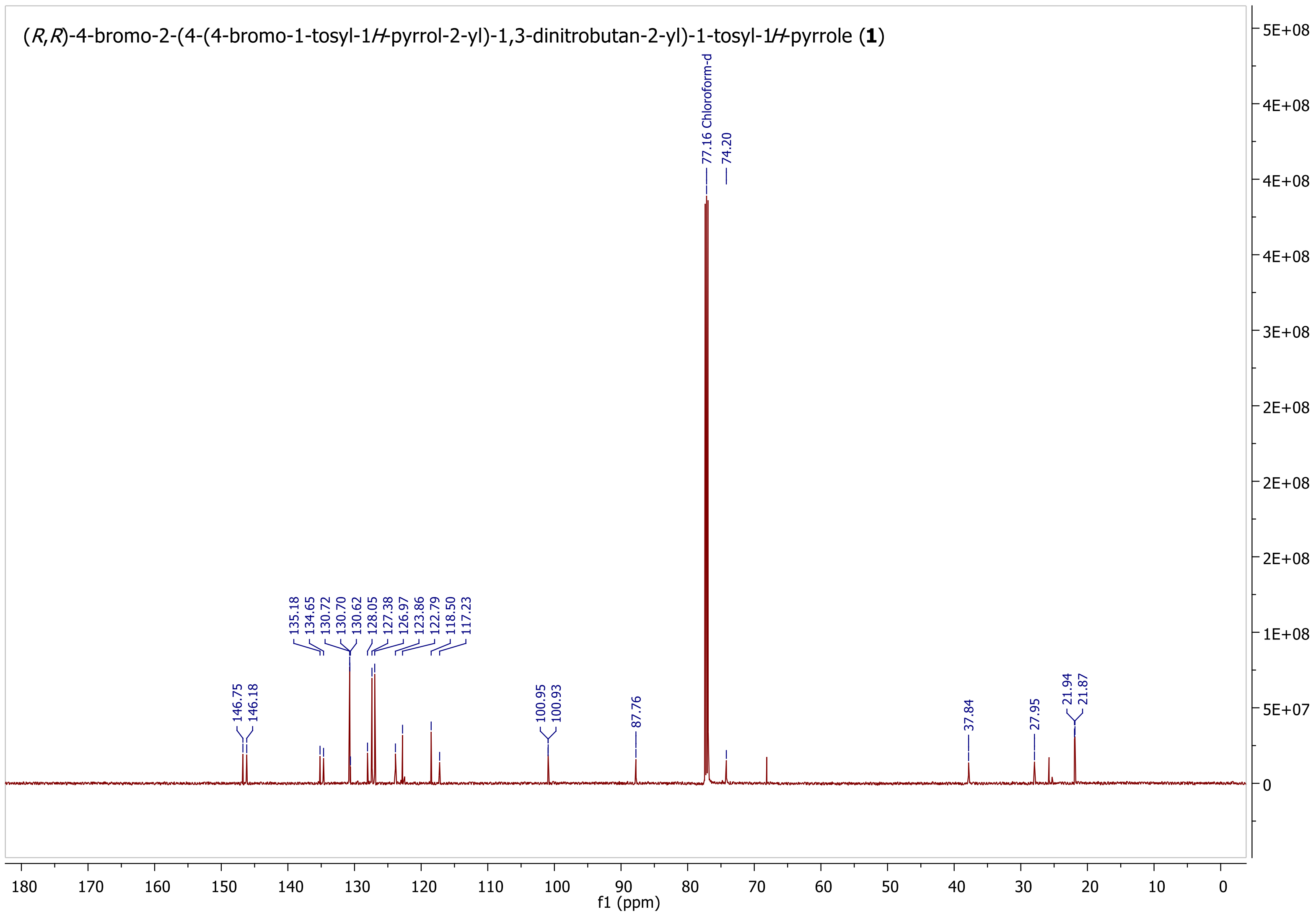

Figure S2. ^{13}C NMR spectra of (*R*,*R*)-4-bromo-2-(4-(4-bromo-1-tosyl-1*H*-pyrrol-2-yl)-1,3-dinitrobutan-2-yl)-1-tosyl-1*H*-pyrrole (**1**) ($CDCl_3$, 151 MHz, 298 K). Chemical shifts were determined with respect to the residual solvent peak.

(*R,R*)-4-bromo-2-(4-(4-bromo-1-tosyl-1*H*-pyrrol-2-yl)-1,3-dinitrobutan-2-yl)-1-tosyl-1*H*-pyrrole (**1**)

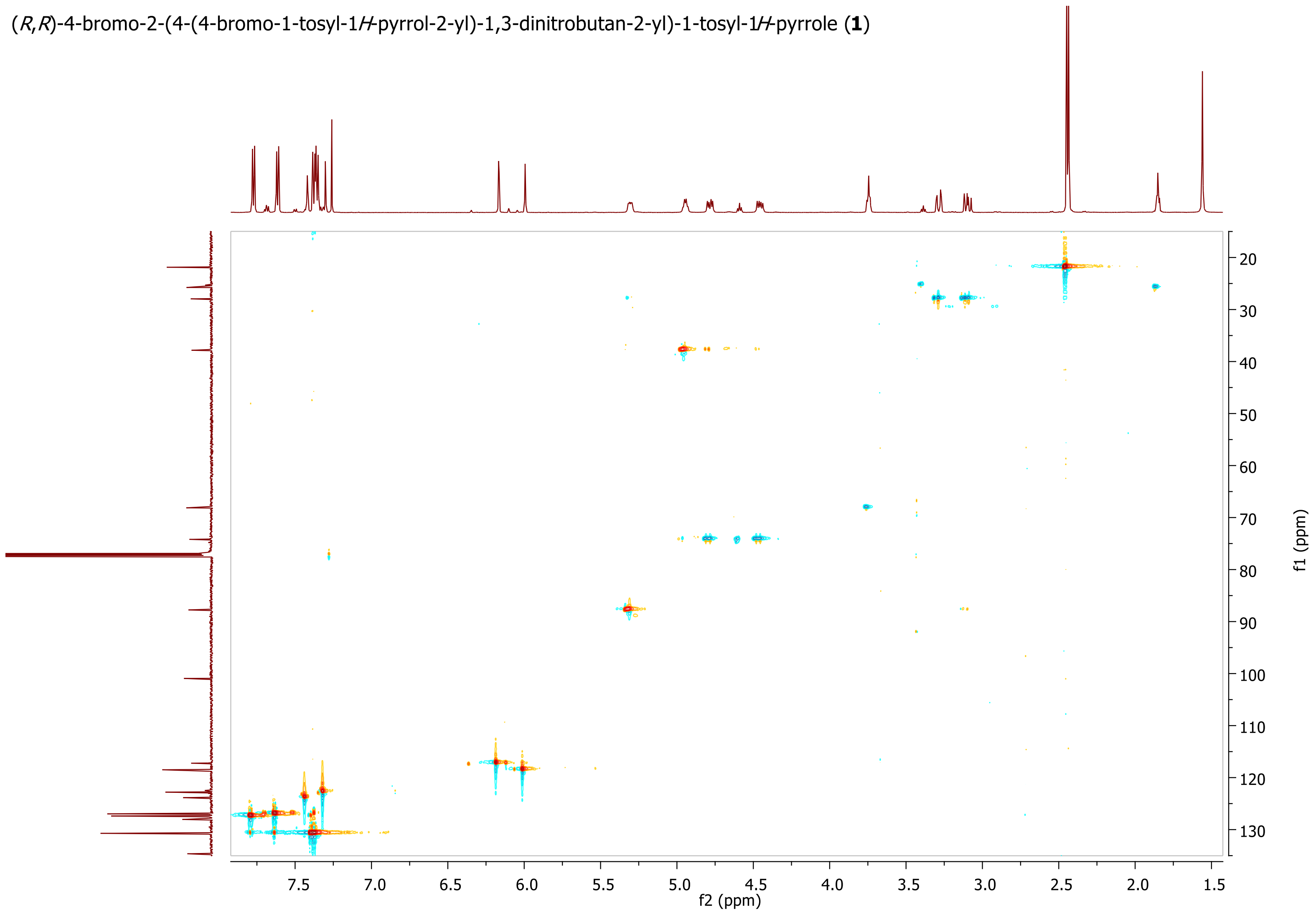

Figure S3. ^{1}H-^{13}C HSQC NMR spectra of (*R*,*R*)-4-bromo-2-(4-(4-bromo-1-tosyl-1*H*-pyrrol-2-yl)-1,3-dinitrobutan-2-yl)-1-tosyl-1*H*-pyrrole (**1**) ($CDCl_3$, 298 K). Chemical shifts were determined with respect to the residual solvent peak. Spectra were aligned using data from the respective ^{1}H and ^{13}C NMR spectra.

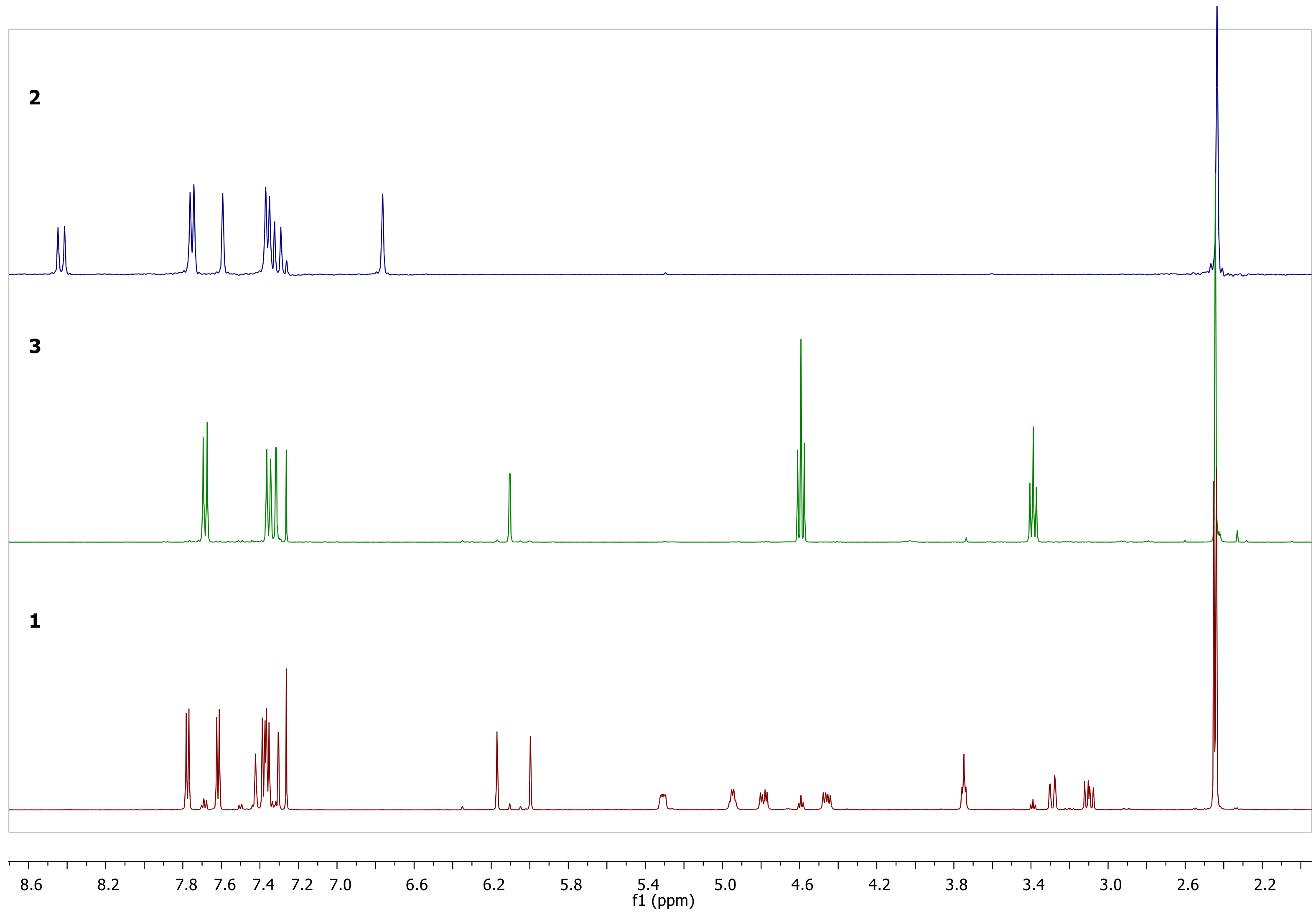

Figure S4. Overlayed ^{1}H NMR spectra of (*E*)-4-bromo-2-(2-nitrovinyl)-1-tosyl-1*H*-pyrrole (**2**), 4-bromo-2-(2-nitroethyl)-1-tosyl-1*H*-pyrrole (**3**) and (*R*,*R*)-4-bromo-2-(4-(4-bromo-1-tosyl-1*H*-pyrrol-2-yl)-1,3-dinitrobutan-2-yl)-1-tosyl-1*H*-pyrrole (**1**) in the range δ = 2.00–8.65 ppm ($CDCl_3$, 298 K).
